# Navigating Role Transition: A Qualitative Study of How New Nurse Managers Develop Resilient Leadership

**DOI:** 10.1155/jonm/8465413

**Published:** 2026-05-30

**Authors:** Ziling Song, Jing Wang, Ping Gao, Xiaoqun Xu, Xiaoxia Huang

**Affiliations:** ^1^ School of Medical Humanities and Management, Wenzhou Medical University, Wenzhou, Zhejiang, China, wmu.edu.cn; ^2^ School of Nursing, Wenzhou Medical University, Wenzhou, Zhejiang, China, wmu.edu.cn; ^3^ Department of Nursing, The First Affiliated Hospital of Wenzhou Medical University, Wenzhou, Zhejiang, China, wzhospital.cn

**Keywords:** new nurse manager, nurse manager, nursing management, qualitative study, resilient leadership

## Abstract

**Aim:**

To explore how new nurse managers develop resilient leadership during early managerial transition and to identify organizational and individual supports that facilitate this process in high‐stress nursing contexts.

**Background:**

Resilient leadership is essential for addressing stress, uncertainty, and workforce instability in healthcare organizations. However, limited research has examined how new nurse managers develop this capability during the early stages of their managerial transition.

**Methods:**

From May to July 2025, a qualitative descriptive study using in‐depth semistructured interviews was conducted with new nurse managers at three Class A tertiary hospitals in Zhejiang Province, China. Data were analyzed using content analysis supported by NVivo 12.0.

**Results:**

Thirteen subcategories were identified and grouped into three main categories: (1) multidimensional challenges in developing resilient leadership; (2) empowering support for developing resilient leadership; and (3) endogenous growth and leadership strategies.

**Conclusion:**

New nurse managers face multidimensional challenges in developing resilient leadership. This capability is shaped through the interaction between individual agency and organizational support and is facilitated by experiential learning, shifts in management philosophy, and team empowerment.

**Summary**
• Implications for nursing management:
◦ Healthcare organizations should move beyond individual‐focused approaches and foster supportive leadership ecosystems to sustainably develop resilient leadership among new nurse managers (NNMs), thereby strengthening team effectiveness and supporting high‐quality care.
• Reporting method:
◦ The study adheres to the Comprehensive Reporting Guidelines for Qualitative Research (COREQ) reporting guidelines.
• Patient or public contribution:
◦ No patient or public contribution.

## 1. Background

Contemporary health care faces numerous challenges, including a shortage of nurses, an aging population, the increasing complexity of care needs, climate change, and limited medical resources [1]. In this high‐pressure environment, nurse managers are among the most critical and demanding roles within healthcare systems worldwide, given the diversity and complexity of their responsibilities [2]. Nurse managers use leadership, adaptability, and decision‐making skills in their managerial roles to coordinate frontline teams, which benefits public health emergency responses. Additionally, their involvement in administrative roles highlights the importance of the nursing perspective in strategic planning, organizational adaptation, and strengthening health system resilience during crises [3]. Despite the strategic importance of the nurse manager position, healthcare leaders still face significant challenges in attracting qualified candidates [4]. This situation mainly results from a severe global shortage of nursing personnel, with projections indicating that by 2030, there will still be approximately 13 million vacant nursing positions worldwide [5]. Furthermore, effectively retaining NMMs is an urgent issue. The added stress of role adaptation, the gap between preemployment expectations and workplace realities, and insufficient preparation often make it difficult for NNMs to meet their responsibilities. Selecting nurse managers usually depends more on clinical experience, seniority, and professional competence than leadership and management skills. Most nurses move into management roles because others recognize their leadership potential rather than actively seeking the position, which may lead to a lack of intrinsic motivation [6]. As a result, many NNMs have had to transition rapidly from direct patient care roles into management positions, developing new competencies such as leadership through trial and error. The WHO’s Global Strategic Directions for Nursing and Midwifery 2021–2025 highlights improving nursing leadership as one of its key strategic priorities [7]. Leadership is how an individual influences and motivates others within an organization or team to reach shared goals [8]. Furthermore, leadership is a multidimensional construct comprising leadership knowledge, skills, and personal attributes (e.g., emotional intelligence), with leadership styles such as transformational and ethical leadership reflecting the practical enactment of leadership in organizational contexts [9]. As vital members of the nursing management team, nurse managers should leverage their leadership knowledge, skills, and attitudes to influence nurses’ attitudes, emotions, and beliefs. This allows for the full implementation of leadership practices to achieve common organizational goals. Effective leadership is closely linked to many positive outcomes and is essential for delivering high‐quality care, increasing patient satisfaction, and ensuring patient safety [10, 11]. Additionally, leadership positively impacts nursing professionals’ work environment, professional identity, and team stability [12].

The development of leadership and the growth of nurse leaders are essential to the success of the nursing profession [13]. As patient and caregiver needs increase, nurse managers face new demands on their management and leadership skills, especially for NNMs encountering greater challenges in their initial managerial roles. NNMs are defined as nurses who transitioned directly from clinical roles to nurse manager positions and had no more than 2 years of managerial experience [14]. However, NNMs often face transition shock, feeling overwhelmed, stretched thin, emotionally drained, and burned out [14]. They typically must handle significant responsibilities and pressure, ethical dilemmas, complex interpersonal relationships, heavy administrative tasks, and conflict resolution—often while overworked and lacking sufficient training or support [15]. Such fragile situations can lead to professional burnout and turnover among NNMs [16], further threatening job satisfaction, nursing staff retention, patient safety, and organizational performance [17, 18]. Interestingly, resilient leadership has been identified as a key capability that enables NNMs to navigate these inherent challenges and crises successfully [19]. This capability integrates emotional intelligence, adaptive leadership behaviors, and personal traits associated with individual resilience. Leaders’ perseverance and capacity to confront adversity have been recognized as critical drivers of sustained organizational development. As the role of resilience in social and managerial practice has become increasingly salient, resilient leadership has attracted growing attention from both academia and the business community [20]. Despite this, the concept of resilient leadership remains vaguely defined, with few studies exploring it in depth. Prior research describes resilient leadership as a mindset that equips leaders to adapt quickly and take strategic action to overcome adversity, thereby facilitating organizational recovery and growth [20]. Resilient leadership among nurse managers refers to the capacity to lead teams in responding to workplace adversity, as well as the personal strengths they draw upon; that is, it represents not only a critical leadership capability but also an individual coping resource that supports role transition, emotional regulation, and the sustained fulfillment of managerial responsibilities [21]. Conversely, nursing leaders with low resilience may struggle to inspire and empower others. In contrast, highly resilient nursing supervisors are more likely to boost staff confidence, prioritize well‐being, identify and leverage strengths, and promote professional growth [22, 23]. Resilient leadership also benefits employee performance, engagement, and overall organizational outcomes [20, 24]. Numerous studies emphasize nurse managers’ work‐related pressures, underscoring resilience as a key personal trait for coping with stress [25, 26]. Given ongoing staffing shortages, the complex roles and responsibilities of nursing supervisors, and their high turnover rates, there is a clear need to understand the resilient leadership necessary for effective performance.

Previous scholars have concentrated their research on the quantitative relationship between nursing leadership styles (such as transformational and ethical leadership) and outcome variables such as nurse burnout [27] and patient safety [28]. As healthcare systems become increasingly complex, the need for capable and resilient leadership has become urgent [29]. However, research on the development of resilient leadership remains limited. Quantitative methods do not fully reveal how NNMs build resilience in specific dilemmas or explore their internal experiences [22, 30]. Their internal struggles and cognitive changes during growth are hard to capture through questionnaire data. Additionally, research has examined various aspects, such as role readiness [14] and organizational support [31], to aid NNMs in their role transition and adaptation. However, no studies have examined how resilience‐based leadership can help these new leaders better manage role adaptation and stress. Many researchers often view nurse managers as a homogeneous group, overlooking NNMs’ unique challenges during their particularly vulnerable transition phase. The opportunities for developing resilient leadership during this period, such as overcoming challenges, gaining empowerment, and fostering growth, have not been sufficiently studied. Against this background, this study uses a descriptive qualitative approach to interview NNMs in general hospitals. Its goal is to explore the challenges these nurse managers encounter in developing resilient leadership during their role transition and natural growth. The research aims to provide empirical evidence to help hospitals create effective resilience‐based leadership development programs for NNMs. Such programs could significantly improve these nurse managers’ role adaptation and professional growth, ultimately boosting nursing management effectiveness and team stability.

## 2. Methods

This descriptive qualitative study used a semistructured interview approach. This design was chosen to obtain rich, practice‐based descriptions of NNMs’ experiences, perceptions, and contextual challenges related to resilient leadership development. A qualitative descriptive approach is particularly suitable for exploring under‐researched phenomena in applied healthcare settings and for producing findings that are directly relevant to management practice. The Institutional Review Board of the author’s hospital approved the study ethically (Ethics Approval No. KY2025‐409).

### 2.1. Respondents

Using purposive and maximum variation sampling strategies, NNMs from tertiary hospitals in Zhejiang Province were selected for in‐depth interviews between May and July 2025. This sampling strategy was adopted to capture diverse perspectives across clinical departments, gender, and managerial contexts, thereby enhancing the credibility and transferability of the findings. NNMs were defined as nurses who transitioned directly from clinical roles to nurse manager positions and had no more than 2 years of managerial experience. The inclusion criterion was NNMs with less than 2 years of managerial experience. Not all nurse managers work in clinical frontline departments. Their leadership responsibilities, work content, and pressures differ from those of nurse managers in clinical units. In addition, during the data collection period, some nurse managers were unable to participate in their work due to off‐site training, sick leave, or other prolonged absences, which limited our ability to elicit resilience‐related information through interviews fully. Therefore, the following exclusion criteria were applied: ① nurse managers not working in clinical frontline departments and ② nurse managers who were unavailable due to off‐site training, sick leave, or other reasons. Eligible participants were identified with assistance from nursing administration departments at participating hospitals. The first author contacted potential participants via email, explained the study purpose and procedures, and addressed any questions. Those who agreed to participate were scheduled for interviews. All participants reviewed and signed written informed consent forms before participation. The sample size was determined by collecting data until no new themes emerged and data saturation was achieved. A total of 20 NNMs were interviewed, labeled N1–N20.

### 2.2. Data Collection

Data were collected through semistructured interviews with NNMs. Prior to the formal data collection, two NNMs were purposively recruited to participate in pilot interviews. Insights gained from the pilot interviews were used to refine and enhance the interview guide. The finalized interview guide was designed to explore in depth NNMs’ experiences with resilience leadership (Table 1). The semistructured interview guide consisted of a set of core questions consistently applied across all interviews, with flexibility for probing and follow‐up questions based on participants’ responses. We specifically inquired about the challenges and supports encountered in developing their resilience leadership. Ultimately, 20 NNMs (3 males and 17 females) participated in this study. The general profile of the study participants is presented in Table 2. After 20 interviews, data saturation was reached as no new themes emerged. Data saturation was determined collaboratively by the research team through ongoing comparison of emerging codes and themes, with consensus reached that no new substantive information was being generated. The first author conducted two additional interviews in late August 2025 to rule out potential new information. Following this, the researchers decided to conclude data collection. Interviews were conducted in Chinese via face‐to‐face meetings or video chat, lasting 30–45 min. The interview sequence and approach were flexibly adjusted based on participants’ responses during communication. Valuable answers were followed up with appropriate probing questions. Responses that contained rich contextual details, revealed critical dilemmas, exemplified adaptive strategies, or expressed strong emotional reflections were identified as particularly valuable for understanding resilience development. These responses were followed up with probing questions (e.g., “Could you please describe in detail a specific situation where you felt under pressure?” or “How did your perspective change after that experience?”) to elicit deeper insights. All interviews were audio‐recorded with participants’ consent, and field notes were taken immediately after each interview to capture contextual information and preliminary reflections. Interviews were transcribed verbatim within 24 h. To protect participant privacy, interview content was anonymized. When preparing this manuscript, the COREQ were followed [32].

**TABLE 1:** Interview outline.

No.	Questions
1	Could you share your biggest takeaway from transitioning from a nurse to a new nurse manager? What has your work experience been like during this period?
2	What are the main difficulties and pressures you encounter in your current work?
3	When facing these challenges, where do you typically turn for support?
4	Looking back on this experience, in what ways do you feel you have changed? What effective coping strategies have you summarized?
5	If you were to develop a resilient leadership development strategy for new nurse leaders, what core sections would you include?
6	Do you have anything to add to the above questions?

**TABLE 2:** Sociodemographics of participants (*n* = 20).

Characteristics	*N* = 20
Age range (years)	34–44
Gender	
Female	17
Male	3
Educational level	
Undergraduate	7
Postgraduate	13
Professional	
Middle rank	17
Senior professional	3
Hospital	
A	9
B	7
C	4

*Note:* A, B, and C represent three different hospitals.

### 2.3. Data Analysis

To ensure data accuracy and completeness, two researchers independently listened to the recordings multiple times after the interviews and transcribed verbatim in Chinese within 24 h of data collection. Identifying information was removed during transcription, and participants were assigned numerical codes (N1–N20) to ensure anonymity. After transcription, the texts were returned to the interviewees for verification to guarantee the stability and accuracy of data collection and research findings. Transcripts were coded sequentially by time (N1, N2, N3, …, N20; 20 codes in total) and imported into NVivo 12.0 for sequential analysis. Coding and analysis were conducted in Chinese to preserve semantic and contextual accuracy, and key quotations were translated into English and checked by a bilingual researcher to ensure conceptual equivalence. Content analysis was used to identify themes. The process includes reviewing the original materials to become familiar with the overall content; analyzing each sentence individually, annotating key statements, and performing open coding; comparing and grouping related codes to gradually form themes and subthemes; and defining themes, subthemes, and codes while selecting representative expressions from the original data. When discrepancies arise during analysis, the research team meets to discuss and review them and refine the final themes.

## 3. Results

Based on the data described in Table 3, we identified three main categories and 13 subcategories. To illustrate NNMs’ perspectives, the following interpretations and direct quotations (Chinese translations) clarify the meaning of each theme.

**TABLE 3:** Main categories and subcategories identified in the study.

Main categories	Subcategories
Multidimensional challenges in developing resilient leadership	System pressure overload
	Insufficient leadership effectiveness
	Interpersonal coordination dilemma
	Persistent physical and mental exhaustion
	Work–family conflict

Empowering support for resilient leadership development	Psychological capital development
	Organizational support system
	Hospital culture empowerment
	Non‐work domain resource support

Endogenous growth and leadership strategy	Learning and imitation
	Transformation of management philosophy
	Mental expansion
	Empowerment through authorization

### 3.1. Theme 1: Multidimensional Challenges in Developing Resilient Leadership

#### 3.1.1. System Pressure Overload

NNMs reported experiencing substantial work pressure arising from institutional performance demands and extensive cross‐departmental coordination, often finding themselves positioned within a “sandwich layer” between administrative expectations and clinical practice.

Formal evaluation systems, including quality indicators, patient safety accountability, and emergency response assessments, were described as major sources of pressure during the early leadership transition. Unclear, and at times shifting, expectations from upper management further contributed to a sense of uncertainty, leaving NNMs in a challenging position between administrative requirements and frontline clinical realities.

One participant reflected, *“I don’t know what it takes to satisfy the boss. Even when you’ve given it everything, it still might not be enough. We’re like a sandwich in the middle, under pressure from both sides”* (N5). Another NNM stated, *“All surgeons are in the operating rooms, leaving patient care completely to residents. Nurses face immense pressure regarding patient safety, especially with constant emergency response evaluations and rankings”* (N7).

Beyond formal performance demands, NNMs also reported that extensive cross‐departmental coordination substantially increased their daily workload. Time was frequently consumed by communication, mediation, and problem‐solving, which limited their autonomy over their work schedules.

One nurse manager explained, *“Every day I deal with complex coordination across departments. I feel like I have no control over my own time, and sometimes I cry, feeling completely alone”* (N11). Another participant similarly described, *“Patient safety quality management, personnel management, and department development are all extremely demanding. The pressure feels endless”* (N14).

#### 3.1.2. Insufficient Leadership Effectiveness

NNMs reported experiencing insufficient leadership effectiveness during their early transition, characterized by limited management preparedness and uncertainty in leading teams and departmental development.

Several participants linked these challenges to the absence of systematic management training and structured onboarding. Entering managerial roles with limited preparation, NNMs described gaps in specialized knowledge and leadership skills.

One participant stated, *“I wasn’t previously part of this department, and my lack of specialized knowledge makes it difficult to lead its development”* (N5). Another compared herself with experienced leaders, noting, *“Some teachers stand there like towering trees, sheltering everyone beneath them. I haven’t grown into a towering tree yet”* (N6).

Beyond individual competence, NNMs also described difficulties in mobilizing teams and advancing departmental planning. Coordinating collective action, encouraging engagement, and establishing clear developmental directions were perceived as challenging during the early stages of leadership.

One nurse manager explained, *“It’s still quite difficult for me to get everyone to work together as a team. I worry about not planning the department well and often feel lost”* (N16). Others reported limited success in motivating subordinates to participate in training programs or professional activities. One participant stated, *“Some nurses are not very willing to take part in competitions or training programs. I find it difficult to motivate them”* (N20).

#### 3.1.3. Interpersonal Coordination Dilemma

NNMs reported experiencing persistent interpersonal coordination difficulties during their leadership transition, involving team integration, cross‐departmental communication, and interactions with both subordinates and superiors.

Several participants described challenges in establishing trust and cohesion within their teams, particularly when managing nurses transferred from other departments. Differences in prior work practices and expectations often led to resistance and prolonged adjustment periods, making team integration more difficult.

*“Some nurses will talk about how things were done in their previous departments. There’s always a transition period, and if trust isn’t built, it becomes very hard to move forward with the work”* (N2).

NNMs also reported difficulties in managing interpersonal conflicts and generational differences among nursing staff. Some participants noted that younger nurses required different communication and management approaches, which increased the complexity of daily coordination.

*“You will encounter difficult nurses, and you need to resolve the situation quickly. Otherwise, they’re like a rotten apple that spoils the whole barrel. Nurses born in the 2000s often have stronger self-awareness and higher expectations of fairness”* (N3).

In addition to internal team issues, cross‐departmental coordination was described as a significant source of stress. Participants reported uncertainty about communication channels and hesitation when initiating contact with other departments, which sometimes delayed problem resolution.

*“Cross-departmental mediation is very difficult. I’m often unsure who to contact, and I hesitate because I don’t know how to phrase things properly”* (N4).

Interactions with superiors were also perceived as challenging, particularly when communication lacked openness or psychological safety. Some NNMs reported difficulty expressing their concerns upward, fearing interruption or dismissal.

*“There are things I haven’t discussed with my superiors. If you hit the key point too directly, they might cut you off, and then it’s hard to make any real progress in communication”* (N9).

#### 3.1.4. Persistent Physical and Mental Exhaustion

NNMs reported experiencing persistent physical and mental exhaustion during their leadership transition, which manifested as prolonged fatigue, psychological distress, and difficulties in achieving adequate rest.

Several participants described significant physical symptoms that they attributed to sustained work pressure. Sleep disturbances, gastrointestinal problems, and stress‐related skin conditions were commonly reported, reflecting the bodily impact of prolonged stress exposure.

One participant stated, *“I often can’t sleep, and I have acne all over my chin. The dermatologist told me it’s stress-related acne”* (N12). Another noted, *“A physical examination showed that I have stomach problems. Poor stomach health is closely related to emotional well-being”* (N5).

In addition to physical discomfort, NNMs reported considerable psychological strain from the constant demands of their role. Participants described feeling persistently anxious and emotionally overwhelmed, particularly when facing unresolved issues or unexpected adverse events.

One nurse manager shared, *“Many problems that can’t be solved are pushed onto the* nurse managers*. It’s exhausting”* (N9). Another participant explained, *“Right after I took over the department, a major adverse event happened. I felt extremely anxious and thought I was going to lose my mind”* (N1).

NNMs also described difficulty separating work from personal life, which further contributed to mental exhaustion. Even during designated rest periods, participants reported remaining mentally engaged with work responsibilities, limiting opportunities for genuine recovery.

One nurse manager explained, *“Even when I want to take a half-day off, I still have to stop what I’m doing and apply for approval through the OA system”* (N9). This constant state of alertness was widely perceived as draining and unsustainable. Moreover, this persistent vigilance often extended beyond the workplace, blurring the boundary between professional responsibilities and personal life and contributing to subsequent work–family conflict.

#### 3.1.5. Work–Family Conflict

NNMs reported experiencing pronounced work–family conflict during their leadership transition, characterized by difficulties balancing professional responsibilities with family roles and personal life demands.

Several participants described limited understanding and support from their spouses, which intensified emotional stress and frustration. The increased workload associated with the nurse manager role often exceeded family members’ expectations, leading to misunderstandings and emotional strain.

*“My husband can’t understand why becoming a* nurse manager *has made me even busier. Sometimes I cry too”* (N3). She further added, *“If I took an anxiety scale test, I would definitely score in the mild to moderate range”* (N3).

Balancing childcare responsibilities with demanding work schedules was also reported as a major challenge, particularly for NNMs with young children. Participants described feelings of guilt and exhaustion resulting from limited time and energy for family care. One nurse manager explained, *“I have two children, one is ten and the other is three. My husband is usually very busy as well. I no longer have time to manage the household, and it feels extremely stressful”* (N5). Another participant shared similar concerns, noting ongoing difficulty in managing both professional and family obligations.

In addition, NNMs reported that work responsibilities frequently extended into personal time, further blurring the boundary between work and home. Even outside formal working hours, participants described remaining psychologically connected to their departments.

*“My nerves are always on edge, worrying about something happening in the department. Even after clocking out, I’m never really off duty. At home, I still log into the cloud desktop to monitor the department”* (N8). She added, *“I don’t have any energy left to take care of my child”* (N8).

### 3.2. Theme 2: Empowering Support for Resilient Leadership Development

#### 3.2.1. Psychological Capital Development

NNMs reported actively drawing on personal psychological resources to cope with work‐related stress, including emotional regulation, cognitive adjustment, and self‐affirmation during challenging situations.

Several participants described consciously managing their emotional states to maintain psychological stability and effective leadership performance. Strategies such as positive self‐talk, deliberate emotional expression, and timely emotional release were commonly mentioned.

One participant stated, *“When I come to work in the morning and feel unhappy, I tell myself to act happy first, and then I really start to feel happy”* (N3). Another explained, *“If anxiety accumulates, it will eventually change in nature. I try to release it early so it doesn’t reach that point”* (N15).

Some NNMs also described using metaphorical thinking and self‐boundary setting to prevent emotional overload. These cognitive strategies helped them recognize psychological limits while maintaining a sense of control.

*“I tell myself there’s a river of anxiety and depression below. I can put my feet in, but I must pull them back quickly. If I fall in, I’ll need someone to rescue me”* (N18).

In addition, a small number of participants reported using personal coping strategies to facilitate emotional relaxation and sleep under intense pressure. One participant stated, *“When the pressure is too much, I drink some sweet fruit wine to make myself a little tipsy, and then I can fall asleep”* (N3).

#### 3.2.2. Organizational Support System

NNMs reported receiving various forms of organizational support that facilitated their adaptation to managerial roles, including trust built on existing relationships, mentorship, and peer support.

Several participants highlighted the importance of relational foundations within the organization, particularly for NNMs promoted internally. Familiarity with colleagues and preexisting trust were described as reducing interpersonal resistance and easing leadership transitions.

*“I’m glad I was promoted locally. If I had been transferred from elsewhere, the pressure would have been twice as much. They trust me and know I’m not doing this for myself, but for the department’s development”* (N3).

Mentorship and guidance from the nursing department were also described as key sources of support. In the participating hospitals, a formal mentoring program was in place for NNMs, whereby each NNM was assigned two mentors to provide guidance during the transition period. Participants reported that these mentors clarified priorities, provided direction when difficulties arose, and supported decision‐making during the early stages of leadership.

One nurse manager explained, *“Both of my mentors have been very supportive. When I encounter problems, they help me find the right direction and guide me on how to handle things properly”* (N10). Another participant noted, *“The leadership supported me, and the nurses under me supported my work. I was able to take over smoothly because the previous* nurse managers *had built a solid foundation”* (N13).

Peer support among NNMs was frequently cited as an important emotional and informational resource. Interacting with colleagues in similar positions helped reduce feelings of isolation and self‐doubt while enabling experience sharing and mutual understanding.

One participant stated, *“Peer support is very important. People at higher levels sometimes think we can’t endure hardship, but those at the same level understand exactly what we’re going through”* (N8). Another shared, *“After talking with people at my level, I realized everyone is in the same situation. It made me feel more confident, knowing I’m not the only one struggling”* (N20).

#### 3.2.3. Hospital Culture Empowerment

NNMs reported that a supportive hospital culture played an important role in facilitating their leadership adaptation by providing institutional assurance and humanistic care.

Several participants described how tangible institutional support from the hospital enhanced their sense of security and organizational belonging. Practical benefits, such as housing support and stable welfare policies, were perceived as alleviating external stress and enabling NNMs to focus more fully on their leadership roles.

One participant stated, *“Over the years, our hospital has done very well in many aspects. If the hospital didn’t provide us with housing, we would have had to rent on our own, and the pressure would be much greater”* (N15).

Humanistic management practices within the nursing department were also frequently mentioned as an important cultural support. Participants noted that efforts to reduce unnecessary evaluations and formalistic activities helped ease workload pressure and improve morale.

*“The Nursing Department really emphasizes humanistic care. For Nurses’ Day this year, they reduced the workload and didn’t include evaluations. That approach truly reflected what everyone hoped for”* (N19).

In addition, responsiveness to frontline concerns was described as a key feature of a supportive hospital culture. When staffing shortages or operational difficulties were raised, timely organizational responses helped NNMs feel acknowledged and supported. *“When we reported staffing shortages, the Nursing Department quickly arranged support from floating nurses. That made us feel heard and supported”* (N17).

#### 3.2.4. Non‐Work Domain Resource Support

NNMs reported drawing on resources from the non‐work domain to cope with work‐related stress, including support from spouses, extended family, and friends.

Several participants emphasized the importance of spousal understanding and cooperation in reducing daily stress and role burden. Emotional support and shared responsibility at home were described as providing reassurance and stability amid demanding work schedules.

One participant stated, *“My husband also works at this hospital, so we understand each other very well. We take care of the children together, which gives me a lot of freedom”* (N2). Another shared, *“My husband often enlightens me. He tells me to use both upward and downward resources and to unite all the forces that can be united”* (N14).

Support from extended family members, particularly older relatives, was also described as an important practical resource. Assistance with childcare and household responsibilities alleviated time pressure and enabled NNMs to manage work demands more effectively.

*“My mother-in-law is retired and helps me take care of the baby full-time. When I have meetings on Saturdays, she also helps keep the house in order”* (N6).

In addition, participants described friendships and social interactions outside of work as meaningful sources of emotional release. Informal activities such as shopping, traveling, and casual gatherings with friends were perceived as helping them relieve stress and regain emotional balance.

*“I have a group of friends who like to have fun together. We go shopping, travel, and joke around, and it really helps me release stress”* (N10).

### 3.3. Theme 3: Endogenous Growth and Leadership Strategy

#### 3.3.1. Learning and Imitation

NNMs reported that actively engaging in learning and imitation were key strategies for improving their leadership competence during the early stages of their managerial roles.

Several participants described seeking formal training and observational learning opportunities to broaden their perspectives and strengthen their management skills. Exposure to external training programs and learning from experienced peers were perceived as essential ways to address initial skill gaps.

*“I really want to attend management training courses and go out to see more, so I can broaden my horizons and think more clearly”* (N15).

Others emphasized the influence of role models within their professional environment. By closely observing the behaviors and decision‐making styles of respected leaders, NNMs reported gradually adopting practical leadership approaches.

*“My previous* nurse managers *were very resilient, emotionally stable, and decisive. I admired them, and after working with them more, I gradually learned their way of handling things”* (N18).

In addition, some participants highlighted learning through reflective guidance from senior colleagues. Honest communication and mentor advice helped NNMs adjust their leadership approach and build credibility with their teams.

One participant shared, *“A senior nurse told me to be honest with the nurses about some of the difficulties and considerations of being a nurse manager. After I did that, they could see that I was sincere”* (N12).

#### 3.3.2. Transformation of Management Philosophy

NNMs reported a gradual shift in their management philosophy during their leadership transition, moving from control‐oriented approaches to more people‐centered, empowering practices.

Several participants described moving away from rigid, task‐driven management styles and toward greater attention to interpersonal relationships and team climate. Creating a relaxed, supportive working environment was perceived as necessary to foster cooperation and reduce resistance.

One participant stated, *“Sometimes I think managing a department is actually like educating children. I’m more willing to use incentives and create a slightly more relaxed atmosphere”* (N15). Another explained, *“I’m not a very demanding leader. I want the team to feel comfortable and live together harmoniously, and I want to get along with them like friends”* (N20).

Others reflected on changes in decision‐making styles, noting increased openness to listening and incorporating staff input. Compared with earlier management practices that emphasized strict compliance, participants reported becoming more inclusive and communicative.

*“In the past, I only cared about setting rules and making everyone follow them, and I often ignored people’s opinions. Now I listen to everyone’s ideas on most things”* (N17).

#### 3.3.3. Mental Expansion

NNMs reported experiencing mental expansion through their leadership roles, reflected in enhanced psychological endurance, broader perspectives, and increased confidence in facing complex situations.

Several participants described that ongoing exposure to managerial challenges contributed to greater psychological strength over time. Although the work was demanding, some NNMs perceived personal mental growth as they learned to cope with pressure and uncertainty.

*“I feel mentally much better than before. Dealing with these work problems has really made me stronger psychologically”* (N5).

Others reported changes in their cognitive perspectives, noting a shift from focusing on individual tasks to considering departmental or organizational interests. This broader outlook influenced decision‐making and the prioritization of responsibilities.

*“Now when I make decisions, I think about what’s best for the whole department. Before, it wouldn’t have mattered so much”* (N13).

In addition, some participants described increased tolerance for uncertainty and a willingness to start over when facing setbacks. Rather than seeking immediate control or certainty, NNMs reported becoming more patient and resilient as they rebuilt their leadership capacity.

*“Now I dare to start over, and I’m willing to take my time to build myself up again”* (N17).

#### 3.3.4. Empowerment Through Authorization

NNMs gradually adopted authorization and delegation strategies as part of their leadership practice, recognizing teamwork and shared responsibility as essential to effective management.

Several participants described a shift from handling tasks personally to delegating responsibilities and coordinating team efforts. Through authorization, NNMs aimed to improve overall efficiency while reducing individual workload.

*“A leader’s role is to plan, delegate, and then coordinate the whole team. It’s impossible for one person to manage everything alone”* (N16).

Others emphasized that authorization did not mean relinquishing responsibility but rather combining delegation with appropriate supervision. Participants reported learning to trust subordinates while maintaining oversight of outcomes.

One nurse manager explained, *“After authorizing others to do the work, we still need to supervise the results. Once we’ve delegated, we can’t say we didn’t know what was happening”* (N20).

In addition, some NNMs described using incentive mechanisms and team‐based activities to encourage initiative and engagement among staff. Recognition of achievements and the creation of supportive communication spaces were perceived as facilitating empowerment.

One participant stated, *“I have a nurse manager’s handbook that includes incentives, such as recognizing nurses who take part in teaching competitions and publicly acknowledging individual achievements”* (N7). Another shared, *“Sometimes we organize sharing or venting sessions so everyone has a space to release stress and reduce work anxiety in a more relaxed way”* (N18).

## 4. Discussion

This study provides an in‐depth understanding of how NNMs develop resilient leadership during the early stages of managerial transition. This study extends prior quantitative research that has primarily focused on associations between leadership styles and outcomes by elucidating how resilient leadership is constructed, constrained, and transformed in practice. Our findings suggest that resilient leadership among NNMs is best conceptualized as a developmental leadership capability that emerges through the interaction of structural conditions, relational positioning, and individual sense‐making, rather than as a stable personal trait.

### 4.1. Structural Constraints and Leadership Competency Gaps in Early Managerial Transition

NNMs in this study experienced substantial systemic stress during role transition, driven by performance demands, staffing shortages, and patient safety accountability, consistent with existing research [4]. However, these stressors exerted a greater impact due to NNMs’ limited authority, role ambiguity, and lack of experiential reference points [14]. This transitional vulnerability, often characterized as “transition shock” [33], undermined leadership confidence and increased susceptibility to emotional exhaustion and fatigue [17, 34].

The frequently described “sandwich layer” dilemma reflects a structural misalignment between accountability expectations and decision latitude. Such misalignment constrains core leadership competencies, including prioritization, boundary management, and strategic judgment, echoing research highlighting the difficulty novice nurse managers face when shifting from technical expertise to system‐level leadership [6, 14]. Insufficient leadership effectiveness reported by NNMs further reflects gaps in formal leadership preparation, particularly in relational coordination, conflict mediation, and long‐term planning [15, 31]. Notably, these reported gaps align with competency domains repeatedly emphasized in hospital management frameworks, including communication, interpersonal influence, strategic planning, and people management. This suggests that early nurse managers may enter the role with predictable competency‐domain mismatches when organizational expectations outpace preparation [35]. These challenges should therefore be interpreted as developmental competency gaps rather than individual performance deficits, reinforcing the need for structured leadership development and staged role preparation [13, 29, 36]. Recent pilot evidence lends empirical support to this recommendation. A role theory‐informed core competency training program for NNMs, designed using the Kemp model, is feasible and systematically targets transitional competency gaps, providing a practical template for staged, competency‐based preparation [37].

In terms of interpersonal coordination, challenges in cross‐departmental communication illustrate the abrupt transition to formal organizational negotiation roles. Without adequate relational authority and specific negotiation skills, early communication setbacks may erode perceived legitimacy and reinforce avoidance behaviors [38]. Persistent physical and psychological exhaustion represents the embodied consequences of prolonged leadership strain [17, 39], although work–family conflict further limits recovery opportunities and weakens external support systems [39]. Collectively, these findings underscore that resilient leadership cannot be sustained through individual coping alone; organizational interventions such as streamlining administrative processes and granting proportionate autonomy constitute foundational conditions for leadership development [31].

### 4.2. Structural and Relational Empowerment as Enablers of Resilient Leadership

Resilient leadership development was facilitated by coordinated support across psychological, organizational, and cultural levels. Resilience emerged not as a self‐contained personal resource but as a capability activated through access to support, trust, and legitimacy. Psychological capital [20, 22] enabled NNMs to regulate emotions and cognitively reframe adversity, consistent with evidence indicating that psychological capital is a protective mechanism against stress and burnout [17, 26]. This emphasis on sustaining leadership through repeated emotion regulation and meaning‐making aligns with Brooks’ conceptual discussion of fortitude as a basis for sustained leadership presence in nursing [40].

Prior exposure to high‐acuity clinical environments appeared to function as experiential scaffolding, strengthening self‐efficacy and adaptive confidence. These internal resources were most effective when reinforced by organizational support. Mentorship and peer support provided interpretive guidance, emotional reassurance, and normalization of transitional challenges, facilitating reflective learning rather than defensive coping [23, 41, 42]. Findings from this study suggest that the formal two‐mentor arrangement implemented in the participating hospitals may be a valuable organizational strategy for supporting NNMs, particularly by clarifying roles, reducing transition‐related uncertainty, and promoting resilient leadership development.

Hospital culture further shaped leadership resilience by influencing psychological safety and organizational identification. Supportive, humanistic management practices, reflecting characteristics of magnet and learning‐oriented cultures, indicate that resilience is cultivated within environments that tolerate uncertainty and temporary inefficiency during transition [43]. Non‐work domain resources, including family and social support, complemented organizational efforts by restoring emotional balance and sustaining motivation [39, 42]. Therefore, nursing management should move toward integrated empowerment ecosystems that align mentorship, peer support, and family‐sensitive policies to translate resilience principles into sustained practice [29, 42].

### 4.3. Developmental Trajectories of Resilient Leadership From Coping to Leading

In addition to external support, NNMs demonstrated proactive strategies that facilitated resilient leadership development. Learning through observation and imitation enabled NNMs to internalize tacit leadership knowledge by modeling emotionally stable and decisive senior leaders, reinforcing evidence that resilient leadership behaviors are socially learned [44].

NNMs also described a transformation in management philosophy from task‐oriented control toward more inclusive and relational leadership approaches. This shift aligns with evidence on transformational and inclusive leadership, which emphasizes psychological empowerment and participation as drivers of engagement and innovation [11, 45]. Such approaches appear remarkably adaptive for managing younger nursing cohorts and mitigating resistance during early transition. Mental expansion and increased tolerance for uncertainty reflected leadership maturation processes. Repeated exposure to ambiguity recalibrated NNMs’ cognitive frames, enabling a transition from reactive coping to anticipatory leadership [24]. Through delegation and authorization, NNMs redistributed responsibility, reduced personal overload, and strengthened collective efficacy [46, 47].

By fostering shared accountability, NNMs redirected attention toward strategic leadership functions. These practices support sustainable team functioning and are associated with improved organizational resilience and patient outcomes [19, 47]. In the context of global nursing shortages, strengthening NNMs’ resilient leadership capabilities may also contribute to leadership retention and workforce stability [48]. To promote this development, nursing departments should implement targeted interventions, such as cognitive‐behavioral training and mindfulness, to enhance emotional regulation [49]. Structured case review sessions can facilitate the sharing of tacit knowledge about conflict resolution [36]. Public recognition of managers who demonstrate strategic thinking in adversity and the use of empowerment‐oriented tools can further clarify growth paths and support professional confidence through constructive feedback [31].

## 5. Limitations

First, the sample was restricted to tertiary hospitals in Zhejiang Province, which might not fully represent broader perspectives. Research on resilient leadership should be expanded into primary care, where cumulative stressors are commonplace and require ongoing management. Second, the study lacked ongoing observation of NNMs’ resilience‐based leadership. Future research could undertake more detailed and thorough investigations to improve understanding and provide a stronger evidence base.

## 6. Conclusion

This study advances understanding of resilient leadership by showing how NNMs develop it during the early stages of their managerial transition. Resilient leadership emerged not as a fixed personal trait but as a context‐dependent capability shaped by individual agency and organizational conditions. Although NNMs face multidimensional challenges, these experiences can also foster growth through experiential learning, reflective shifts in management philosophy, and team empowerment. Healthcare organizations should therefore move beyond individual‐focused approaches and build supportive leadership ecosystems, including structured mentoring models such as the formal two‐mentor system. Selection and training should also emphasize intrinsic motivation and reflective capacity to support sustainable, resilient leadership, leadership stability, team effectiveness, and healthcare outcomes.

## 7. Implications for Future Nursing Management Research

Our research uncovers the challenges, support systems, and growth pathways for developing resilient leadership among NNMs. It also shows that cultivating resilient leadership is crucial for helping these leaders successfully transition into their roles and avoid professional burnout. Additionally, it is vital to build resilient nursing teams capable of responding effectively in high‐pressure environments and ensuring patient safety. This creates a strong foundation for improving the healthcare system’s overall resilience and nursing quality, highlighting key directions for future in‐depth research and developing resilience leadership programs for NNMs. Future research should further examine how mentoring programs, supportive hospital culture, empowerment strategies, and system‐wide organizational supports shape the development and sustainability of resilient leadership among NNMs, particularly in relation to role adaptation, leadership confidence, and long‐term retention.

## Author Contributions

Ziling Song conceived the study, analyzed the data, collected the data, prepared figures and/or tables, and authored or reviewed drafts of the paper. Jing Wang conceived the study, analyzed the data, and authored or reviewed drafts of the paper. Ping Gao collected the data, prepared figures and/or tables, and authored or reviewed paper drafts. Xiaoqun Xu and Xiaoxia Huang conceived the study, audited the initial analyses and interpretation, and authored or reviewed drafts of the paper.

## Funding

No funding was obtained for this research.

## Disclosure

All authors approved the final draft.

## Conflicts of Interest

The authors declare no conflicts of interest.

## Data Availability

The data supporting the findings of this study are available from the corresponding author upon reasonable request and are not publicly available due to privacy or ethical restrictions.
